# Mesenchymal stem cells: environmentally responsive therapeutics for regenerative medicine

**DOI:** 10.1038/emm.2013.94

**Published:** 2013-11-15

**Authors:** Matthew B Murphy, Kathryn Moncivais, Arnold I Caplan

**Affiliations:** 1 Celling Biosciences, Austin, TX, USA; 2 Department of Biology, Skeletal Research Center, Case Western Reserve University, Cleveland, OH, USA

**Keywords:** anti-apoptotic, anti-inflammatory, antimicrobial, mesenchymal stem cell, pericyte, trophic

## Abstract

Mesenchymal stem cells (MSCs) are partially defined by their ability to differentiate into tissues including bone, cartilage and adipose *in vitro*, but it is their trophic, paracrine and immunomodulatory functions that may have the greatest therapeutic impact *in vivo*. Unlike pharmaceutical treatments that deliver a single agent at a specific dose, MSCs are site regulated and secrete bioactive factors and signals at variable concentrations in response to local microenvironmental cues. Significant progress has been made in understanding the biochemical and metabolic mechanisms and feedback associated with MSC response. The anti-inflammatory and immunomodulatory capacity of MSC may be paramount in the restoration of localized or systemic conditions for normal healing and tissue regeneration. Allogeneic MSC treatments, categorized as a drug by regulatory agencies, have been widely pursued, but new studies demonstrate the efficacy of autologous MSC therapies, even for individuals affected by a disease state. Safety and regulatory concerns surrounding allogeneic cell preparations make autologous and minimally manipulated cell therapies an attractive option for many regenerative, anti-inflammatory and autoimmune applications.

## Introduction

Mesenchymal stem cells (MSCs) have been suggested to be patient-specific drugstores for injured tissue, and with good reason.^1^ What was originally believed to be a simple differentiation or lineage cascade of mesenchymal tissue cells has proven itself to be a much more elegant and complex entity. MSCs are now known to originate as pericytes, which function as surveyors of their kingdoms, capable of responding to local environmental stimuli with a myriad of beneficial interventions.^2^ The availability and versatility of these remarkable cells make them an excellent treatment option for a wide variety of clinical pathologies, and it falls to the scientific community to establish clear guidelines for the optimal administration of MSC-based therapies. In the following review, we survey a brief history of MSCs, their anti-inflammatory, immunomodulatory and paracrine effects, and the current status of MSC-based therapies for a multitude of clinical applications.

## Identification and biology of MSCs

### Origin and early research

The presence of regenerative cells was first hypothesized in the late nineteenth century by Cohnheim,^3^ who believed bone marrow (BM)-derived fibroblasts were involved in wound healing throughout the body. After the turn of the century, others described a fundamental relationship between developing mesoderm tissue and blood during embryogenesis.^4^ In the 1960s and 1970s, Friedenstein *et al.*^5, 6, 7^ described the isolation of stromal cells from BM by plastic adherence, a clonal or colony-forming capacity (that is, CFU-F), and the ability to regenerate or support ectopic bone, stroma and hematopoietic tissues. In the late 1980s and early 1990s, the heterogeneous population of MSC from BM was explored and found to be linked to the development of various mesenchymal tissues, as well as identifying the first surface antigens expressed by MSC (cluster of differentiation (CD)73 and CD105).^8, 9, 10, 11, 12, 13, 14^ Because of their linkage with the formation of mesenchymal tissues during embryonic development, these cells were termed ‘MSCs.'^15^

### Tissue sources of MSC

After their initial discovery in BM, MSCs have been isolated and characterized from several adult and fetal tissues, including adipose (fat), dermis (skin), synovial fluid, periosteum, umbilical cord blood, placenta and amniotic fluid. The reported MSC frequency (as measured by CFU-F) and native concentration from several adult human tissues are reported in Table 1. The relative abundance of MSCs throughout the body is understandable in light of recent findings that most, if not all, MSCs are of perivascular origin.^16, 17, 18, 19, 20^ Furthermore, there is a direct correlation between MSC frequency and blood vessel density in stromal vascularized tissue.^21^ MSCs and pericytes share the phenotypic surface markers melanoma cell adhesion molecule (CD146) and platelet-derived growth factor receptor.^16, 22^ It is hypothesized that pericytes are the *in vivo* source of MSCs, with cellular components protruding into the endothelial lumen of blood vessels to monitor and react to systemic signals.^23, 24, 25, 26^ The widespread distribution of perivascular precursors for MSCs would account for their ability to respond to injury by sensing and secreting chemokines locally in response to injury, infection or disease in all vascularized tissues of the body.^1, 27, 28, 29^

### Capacity of MSC

#### Trophic properties of MSC

The primary trophic property of MSCs is the secretion of growth factors and other chemokines to induce cell proliferation and angiogenesis. MSCs express mitogenic proteins such as transforming growth factor-alpha (TGF-α), TGF-β, hepatocyte growth factor (HGF), epithelial growth factor (EGF), basic fibroblast growth factor (FGF-2) and insulin-like growth factor-1 (IGF-1) to increase fibroblast, epithelial and endothelial cell division.^30, 31, 32, 33^ Vascular endothelial growth factor (VEGF), IGF-1, EGF and angiopoietin-1 are released to recruit endothelial lineage cells and initiate vascularization.^34^ It has been hypothesized that an individual's genotype has a role in the expression of and reaction to these cytokines, providing credence to the philosophy of personalized medicine utilizing responsive agents (that is, MSCs) rather than a dose of recombinant proteins or autologous growth factors (for example, platelet-rich plasma).^35^ The trophic effects extend beyond cell proliferation to the reduction of scar tissue formation presumable by local cells secreting paracrine factors keratinocyte growth factor, stromal cell-derived factor-1 (SDF-1) and macrophage inflammatory protein-1 alpha and beta.

#### Anti-inflammatory and immunomodulatory properties of MSC

In many types of musculoskeletal trauma, inflammatory conditions at the site of injury impede the natural repair processes by local progenitor and mature cells. MSCs assist via paracrine mechanisms and modulate the regenerative environment via anti-inflammatory and immunomodulatory mechanisms. In response to inflammatory molecules such as interleukin-1 (IL-1), IL-2, IL-12, tumor necrosis factor-α (TNF-α) and interferon-gamma (INF-γ), MSCs secrete an array of growth factors and anti-inflammatory proteins with complex feedback mechanisms among the many types of immune cells (Table 2).^36, 37, 38, 39, 40, 41^ The key immunomodulatory cytokines include prostaglandin 2, TGF-β1, HGF, SDF-1, nitrous oxide, indoleamine 2,3-dioxygenase, IL-4, IL-6, IL-10, IL-1 receptor antagonist and soluble tumor necrosis factor-α receptor. MSCs prevent proliferation and function of many inflammatory immune cells, including T cells, natural killer cells, B cells, monocytes, macrophages and dendritic cells.^37, 42, 43^ Although MSCs across species are able to regulate T-cell activity, the mechanisms are not identical across mammalian species.^44, 45^

A characteristic of chronically inflamed environments is a persistent imbalance in the types of helper T cells and macrophages.^46, 47, 48^ MSCs indirectly promote the transition of T_H_1 to T_H_2 cells by reducing INF-γ and increasing IL-4 and IL-10.^36, 49^ The restored T_H_1/T_H_2 balance has been shown to improve tissue regeneration in cartilage, muscle and other soft tissue injuries, alleviate symptoms of autoimmune diseases and have an anti-diabetic effect.^50, 51, 52, 53, 54^ Similarly, reduction in INF-γ and secretion of IL-4 promotes a shift in macrophages from M1 (pro-inflammatory, anti-angiogenic and tissue growth inhibition) to M2 (anti-inflammatory, pro-remodeling and tissue healing) type, an effect required for skeletal, muscular and neural healing and regeneration.^46, 52, 55, 56, 57, 58^

Undifferentiated MSCs express low to medium levels of human leukocyte antigen (HLA) Class I and low levels of HLA Class II to avoid recognition by the immune system.^59, 60^ This property gives donor MSCs a so-called ‘stealth' ability to go undetected by a host immune system in allogeneic therapies. However, Class I antigen is present at detectable levels and Class II antigen expression can be induced by INF-γ.^61, 62^ Several cases of allogeneic MSC rejection and chronic immune responses have been reported in animal studies and human clinical trials.^63, 64, 65, 66^

#### Anti-apoptotic properties of MSC

Another important property of MSCs is the ability to rescue apoptotic cells induced by traumatic exposures to hypoxia, chemicals/acidity, mechanical damage and radiation. For example, MSCs have proved to reverse apoptosis in cardiomyoblasts after ischemia, as well as damaged neurons and lung fibroblasts.^67, 68, 69^ The anti-apoptotic mechanisms of MSCs are not fully understood, but several key proteins have been identified. IGF-1 and IL-6 secretion increases the expression of Akt (protein kinase B) and NF-κB (nuclear factor kappa-light-chain-enhancer of activated B cells).^70, 71^ Elevated Akt increases secretion of secreted frizzled-related protein 2, a member of the Wnt signaling pathway and a key mediator of anti-apoptosis in fibroblast-like cells.^38, 72, 73, 74^ Block *et al.*^75^ isolated stanniocalcin-1 as an essential molecule for apoptotic reversal in fibroblasts damaged by ultraviolet radiation and acidity. It is unclear if secreted frizzled-related protein 2 and stanniocalcin-1 are linked or independent in their anti-apoptotic mechanisms. It is known that the anti-apoptotic effects causes increased expression of Bcl-2, Bcl-xL and heat shock protein.^76, 77^ In hypoxia-related apoptosis, it is reported that vascular endothelial growth factor secretion by MSCs is significantly increased, and VEGF, HGF and TGF-β1 have a role in reversing apoptosis of endothelial cells.^78^

#### Antimicrobial properties of MSC

**Figure 1:**
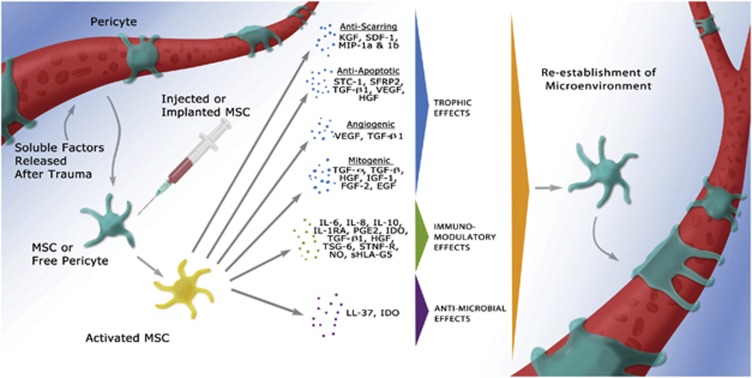
Pericytes are stimulated by soluble growth factors and chemokines to become activated MSCs, which respond to the microenvironment by secreting trophic (mitogenic, angiogenic, anti-apoptotic or scar reduction), immunomodulatory or antimicrobial factors. After the microenvironment is re-established, MSCs return to their native pericyte state attached to blood vessels.^1^

The native immune defense against microbial infections includes effector molecules such as antimicrobial polypeptides, such as cathelicidins, lysozymes, lactoferrin and defensins.^79, 80, 81^ A particular peptide of the cathelicidin family in humans is hCAP-18/LL-37. LL-37 is commonly secreted by epithelial cells and phagocytic macrophages to fight Gram-positive and Gram-negative bacterial infections, but it is also expressed by MSCs.^82, 83, 84^ LL-37 production is a systemic control against bacteria and sepsis, with MSCs secreting the peptide in response to *Escherichia coli* and other microbes.^85, 86, 87^ This discovery suggests that MSCs are a potential therapeutic agent for acute and systemic infections. Bonfield *et al.*^88^ reported that systemic administration of MSCs significantly reduced weight loss, chronic infection, circulating neutrophils relative to macrophages and lung pathologies in a murine cystic fibrosis infection and inflammation model. This study not only demonstrates a direct effect on microbes but also has an influence on immune cell recruitment. It was also observed that human, but not rodent, MSCs combat bacteria and protozoal parasites by the upregulation of indoleamine 2,3-dioxygenase, previously shown to regulate T-cell activity.^38, 39, 89, 90^ The trophic, immunomodulatory and antimicrobial effects of MSCs are illustrated in Figure 1.

### Phenotypic characterization of MSC

After the discovery and early characterization of MSCs, scientists desired a method to prospectively isolate progenitor cells from bulk populations based upon positive or negative selection of CD markers expressed by the cells. The first markers unquestionably identified on MSCs were CD73 (SH-3/4) and CD105 (endoglin or SH-2), followed thereafter by CD90 (Thy-1) and CD44.^13, 91^ It since has been discovered that the quadruple-positive population of CD90^+^/CD105^+^/CD73^+^/CD44^+^ is common to fibroblasts and stromal cells, and only serves to discriminate these cell types from those of hematopoietic origin.^26, 92, 93, 94^ Significant MSC phenotypic characterization has been published in the interim, but unfortunately there remains no strict consensus among the field. In 2006, the International Society of Stem Cell Research established a minimum set of criteria for defining MSCs as: (1) plastic-adherent cells; (2) capable of tri-lineage (bone, cartilage and fat) differentiation; (3) phenotypically positive for CD105, CD73 and CD90; and (4) negative for CD45, CD34, CD11b, CD14, CD79a and HLA-DR.^59^ However, these criteria are based on the characterization of *in vitro* cultured cells and do not apply to the native *in vivo* phenotype. For example, CD34 is considered a marker for hematopoietic stem cells and endothelial progenitors for freshly harvested cells in BM aspirate, but not MSCs.^16, 22^ Others have categorized MSCs harvested from lipoaspirate to be CD34^dim^ or CD34^+^ before *in vitro* culture.^95, 96, 97^ Mitchell *et al.*^98^ demonstrated that 60% of CFU-F-producing cells from the fresh stromal vascular fraction of lipoaspirate are CD34^+^ and that CD34 expression diminishes dramatically with each passage in culture. It was proposed by Zimmerlin *et al.*^19^ that two distinct perivascular cell types from adipose, pericytes (CD146^+^/CD34^−^/CD31^−^) and supra-adventitial stromal cells (CD146^−^/CD34^+^/CD31^−^) give rise to CFU-F. Similar findings of varied primary and cultured phenotypes were reported for MSCs harvested from dermis tissue.^99^ The nonconformity of MSC CD marker expression between the tissues is reasonable based on the potential differences in the perivascular microenvironment of the various tissues in the body. The Simmons lab identified the antibody Stro-1 as a marker to enrich CFU-F more than 100-fold in fresh BM and to identify stromal and osteogenic progenitors.^100, 101, 102^ Stro-1 has been demonstrated on CFU-F derived from stromal vascular fraction cells and localized on the endothelium of some interstitial blood vessels *in vivo*.^103^ Given these findings and the pericyte origin of MSCs, the plausible *in vivo* phenotype of MSCs is CD146^+^/Stro-1^+^/CD90^+^/CD105^+^/ CD73^+^/CD44^+^/CD45^−^/11b^−^/CD14^−^ with little to no expression of CD34. Further prospective research using freshly isolated cell populations from multiple tissues will be required before a consensus on a ‘holy grail' phenotype is reached.

## Clinical translation of MSC therapies

### MSC in orthopedics and spine therapies

#### Fracture repair

It has long been established that osteoprogenitor cells originate in BM and are capable of forming ectopic bone when transplanted.^5, 104^ This property has been clinically exploited in the development of a minimally invasive treatment for non-union fractures.^105, 106, 107^ In a 2005 study using a small volume of injected, unconcentrated, autologous BM aspirate achieved union in 75% of the 20 patients treated within 14 weeks of treatment.^105^ The small volume was chosen so that patients would not experience the ‘pain of suction' associated with larger volumes of BM harvest. In a larger study, 300 ml of BM aspirate (BMA) was collected and concentrated down to 50 ml before injection into non-union fractures.^106^ Out of 60 total patients, 53 (88%) achieved union within 4 months after treatment. The CFU-F analysis of those patient samples revealed the beginnings of an optimal dose range: patients receiving <30 000 CFU-F failed to form a union while those receiving an average of ∼55 000 CFU-F achieved union. Though Hernigou's work alone cannot be considered comprehensive enough to definitively prescribe an effective dose of progenitor cells, it does establish a reasonable guide for early adopters of this type of therapy. BM concentration is an obvious method of increasing the number of progenitors in a small space, but *ex vivo* culture expansion can achieve the same goal. In a small study of six patients receiving injections of culture-expanded, autologous, BM-derived MSCs, four out of five patients seen in follow-up achieved union between 5 and 14 months post procedure, and the non-healing patient's fracture was 40 years old.^107^ An average of 30.25 million MSCs were administered to each patient in this study, which is substantially higher than the numbers used in the Hernigou study. As the Centeno case series is such a small sample, it is difficult to draw any firm conclusions about efficacy. If the success percentages of expanded MSCs bear out in larger studies, the efficacy of culture-expanded MSCs for treatment of non-union fracture could be lower than that of simple concentrated BM aspirate. In that case, concentrated BM presents a number of advantages over expanded cells. Concentrated BM can be used at the point-of-care, in a single surgical procedure, without the risks, cost or time expense of expanding cells in the lab.

#### Osteonecrosis

According to the National Institutes of Health, most osteonecrosis patients will eventually need surgery.^108^ Thus, cell therapy treatments capable of arresting or reversing the progression of osteonecrosis logically pose an attractive alternative to traditional treatments like core decompression, osteotomy and total joint replacement. Gangji *et al.*^109^ treated 13 patients, with a total of 18 stage I or II ostenecrotic hips. Control hips received core decompression, and test patients received core decompression with implantation of autologous BM concentrate (BMC) group; patients and assessors were blinded to treatment group assignment. Patients treated with BMC received an average of 18 400 CFU-F in 51 ml of BMC. At 24 months post procedure, BMC patients had a statistically significant reduction in pain and joint symptoms, while five out of eight patients in the control group had progressed to stage III osteonecrosis. Only one out of ten BMC patients had progressed to stage III in the same time period. A larger study of 56 osteonecrosis patients treated with an average of 31 000 CFU-F in 50 ml of concentrated BM found a 50% reduction in size of the osteonecrotic lesion at 10 years post treatment.^110^ Fifteen hips demonstrated complete resolution of osteonecrosis on magnetic resonance imaging (MRI), and only 7 out of 56 hips had evidence of collapse at 5-year follow-up. Cell therapy options for osteonecrosis are especially attractive for patients with comorbidities like sickle cell disease, for whom joint replacement poses a higher risk of complications and failure.^110^

#### Spine fusion

One of the goals of all spinal fusion surgeries is to grow bone in place of a diseased or damaged intervertebral disc. Iliac crest autograft is the ‘gold standard' technique for achieving this goal, but it is plagued by pseudoarthrosis and donor site morbidity.^111, 112^ Despite the obvious nature of cell therapy for spinal fusion, to our knowledge there is only one peer-reviewed study on the use of cell therapy for spinal fusion. Forty-one patients scheduled for posterior spinal fusion with transpedicular spinal implantation received BMC and beta-tricalcium phosphate graft material.^113^ A volume of 252 ml of BM was harvested from each patient and concentrated to 45 ml of BMC, from which patients received an average of 38 925 CFU-F. A total of 95.1% of patients fused, a superior result as compared with previously reported fusion rates of 65–95% using iliac crest bone graft.^111, 112^ With high fusion rate and lack of donor site morbidity, these results establish BMC as a legitimate and possibly superior alternative to iliac crest bone graft.

#### Cartilage repair

Perhaps owing to the treatment challenge posed by cartilaginous injuries, cellular therapy has been studied rather extensively for cartilage repair. A recent animal model of cell therapy for cartilage repair treated collagenase-induced tendinitis with culture-expanded, autologous BM-derived MSCs.^114^ Histology scores for MSC-treated tendons were statistically significantly more normal than control tendons at the 8-week study end point; tendon stiffness was improved in the MSC-treated tendons but not statistically significantly. A later study in a minipig model compared the efficacy of culture-expanded BM MSCs versus non-expanded BM nucleated cells (BMNCs) with collagen II gel substrates for treatment of full-thickness chondral defects.^115^ Both the MSC and BMNC groups demonstrated statistically significantly improved histology scores over control and substrate alone groups at both 4 and 8 weeks. No statistical difference was found between the MSC and BMNC groups, which suggests the clinical feasibility of point-of-care application of this type of cell therapy.

In 2010, a small study investigated the application of autologous, expanded, BM-derived MSCs for full-thickness articular cartilage defects in five patients. Autologous BM MSCs were expanded, placed in platelet-rich fibrin glue, transplanted into full-thickness cartilage defects and covered with an autologous periosteal flap.^116^ All patients experienced symptom improvement over the course of 12 months post procedure, and the two patients who consented to arthroscopy had nearly normal ICRS arthroscopic scores. A 12 month follow-up MRI of three patients demonstrated complete defect fill and surface congruity; the other two patients demonstrated incomplete surface congruity. Because this study did not include a control group, it is difficult to make an assessment on the superiority of this technique over others. Regardless, the success of this intervention demonstrates both the safety and the possible efficacy of cell therapy for cartilage repair. An interesting permutation of this cell therapy idea was published by Gigante *et al.*^117^ in 2012 in which a patient with knee pain and heterogeneous cartilage (based upon MRI) was treated with an injection of BMC. A volume of 60 ml of BMA was concentrated to 4 ml of BMC, placed in fibrin glue and injected into the debrided joint. The patient had no pain while running at 6 months post procedure, and the 12 month follow-up MRI showed full defect filling. The patient was also asymptomatic at 24 months post procedure.

Most recently, Buda *et al.*^118^ completed a study of 30 patients with osteochondral lesions of the knee. A volume of 60 ml of BMA was concentrated to 6 ml of BMC and soaked onto a hyaluronic acid (HA) or collagen membrane. The BMC containing substrate was arthroscopically implanted into the defect and covered with a layer of platelet-rich fibrin. Mean International Knee Documentation Committee score improved from ∼30 pre-procedure to ∼85 at 29 months post procedure. Knee injury and Osteoarthritis Outcome Score likewise improved from ∼35 pre-procedure to ∼87 at 29 months post procedure. The two biopsied specimens showed regenerated, regularly organized cartilage with homogenous cell distribution. As the authors note, the number of cases completed using this technique is not yet sufficient enough to make sweeping statements about efficacy or indications, but it is sufficient to establish this technique as one of the options in the cartilage repair paradigm.^118^

#### Arthritis

Much like osteonecrosis, osteoarthritis (OA) presents a challenge to the clinician as a progressive, degenerative disease for which there is no clear, effective treatment. Nonsteroidal anti-inflammatory drugs and physical therapy can be effective for early stages of OA, but there are currently no approved treatments known to arrest or reverse progression of the disease. Because inflammation is a known component of OA, even in the earliest stages of the disease, researchers have investigated the use of the inflammation-modulating properties of MSCs for OA pain relief.^119^ Pak^120^ investigated the use of adipose-derived MSCs in the treatment of persistent OA pain. Two patients with a history of unresolved OA pain underwent lipoaspiration to procure adipose-derived MSCs. Lipoaspirates were digested in collagenase then triple washed in 5% dextrose in lactated Ringers solution to ensure removal of all collagenase. The resultant cell suspensions were mixed with HA, a nanogram dose of dexamethasone and activated platelet-rich plasma (PRP) before injection into the symptomatic knee(s). The authors note that patients were instructed to remain immobile for 30 min after cell injection to allow for robust cell attachment. One patient experienced >30% total reduction in visual analogue scale (VAS) score at 12 weeks post treatment, and the other patient demonstrated 86% total reduction in VAS score at 12 weeks post treatment. MRIs for both patients indicated cartilage regeneration at 12-week follow-up. Though only two patients were treated, this study highlights several of the challenges of clinical cell therapies. Pak^120^ used dexamethasone to encourage cartilaginous differentiation. No mention was made of cell counts, so it is difficult to compare cell numbers or characteristics with other studies, even in different applications. In addition, Pak used pre- and post-procedure MRIs as a measure of cartilage growth, but pointed out that it is difficult to find perfectly analogous images. Three-dimensional renderings of the pre- and post-procedure MRIs would make a better case for cartilage regeneration. Despite these shortcomings, it was reported that all of Pak's OA patients indicated varying degrees of pain relief.

In 2011, Davatchi *et al.*^121^ presented the results of a four patient study in which autologous, expanded, BM-derived MSCs were injected into knees of patients with severe OA. Patients received 8–9 million cells in a single injection into the symptomatic knee. For all patients, the walking time to pain, the number of stairs they were able to climb and the VAS score improved after treatment. The authors noted that their results may have been suboptimal as a result of excluding platelets and hematopoietic stem cells from the injectate. This theory could be partially substantiated by looking at the results of similar studies using BMC or BMNCs; however, at this time we are aware of no such studies.

Most recently, Koh and Choi^122^ obtained MSCs from the infrapatellar fat pad and applied them via injection to patients with knee OA. Infrapatellar fat pads were removed from 25 patients undergoing arthroscopic surgery for synovectomy, debridement or cleanup of soft tissue tears or osteophytes. Infrapatellar fat pads were processed by collagenase digestion and centrifugation, and an average of 1.89 × 10^6^ cells suspended in 3 ml of PRP were injected into patients' symptomatic knees. The authors indicate that the cells injected were stem cells but did not specify the characterization method used to verify their status as ‘stem' cells. Study patients also received two injections of PRP into the treated knees at 7 and 14 days after the initial injection. Patients in both the study and the control group experienced improvements in Lysholm score, Tegener activity scale and VAS scores; there was no statistical difference between the control and study groups. The study group had poorer scores initially and more substantial improvements than the control group by the last follow-up. Each of the preceding studies has shown pain relief as a result of a cell therapy intervention, but none of them investigated the effect of dose. Future studies must incorporate cell characterization and dose studies to establish a therapeutic dose range.

#### Disc injection

Degenerative disc disease (DDD) is a progressive condition for which there are currently no effective treatments. Patients are often prescribed physical therapy, exercise and nonsteroidal anti-inflammatory drugs in the early stages of treatment. Conservative treatments are effective for the majority of patients; however, a significant population is left with unresolved pain and more invasive surgical treatments as their only other option. Because the progression of DDD correlates with a decrease in the number of viable cells in the disc, it has been suggested that replacement or augmentation of disc cells may be a viable treatment option for DDD.^123, 124^ Ganey *et al.*^124^ tested this hypothesis in a canine model of disc degeneration. Experimentally degenerated discs were treated with autologous adipose-derived cells in HA, HA alone or nothing. Cell-treated discs had significantly improved matrix translucency, annulus compartmentalization, nucleus pulposus cell density and collagen II and aggrecan synthesis as compared with HA or control.

As the canine spine is structurally substantially different from that of the human spine in both size and orientation, application of this concept to a human model has been slow. In 2011, a small study was completed using autologous, expanded MSCs to treat ten patients' degenerated lumbar discs without annular tears. Each disc was injected with 5–10 million MSCs, resulting in substantial improvements in pain scores and disability. It is notable that pain scores and disability were tracked at multiple time points, revealing that 85% of maximum pain relief was attained by the 3-month time point. This application of MSCs takes advantage of the special properties of MSCs. Their anti-inflammatory capacity may be responsible for the early stages of pain relief, whereas their trophic effects and differentiability may be responsible for slower, more long-term decline in pain and disability.

### MSC in cardiovascular therapies

#### Cardiac

Myocardial infarction is a multi-faceted insult to the cardiovascular system, stemming from the initial ischemic event; the extent of damage and subsequent cardiac disease correlates with the size of the original infarcted region.^125^ Frantz *et al.*^126^ have proposed the possibility of anti-inflammatory agents for minimization of deleterious post-myocardial infarction tissue remodeling. Several clinical studies have recently investigated the use of MSCs for this purpose; however, there has been no consensus yet on preferred delivery method or type of cell. In a randomized, placebo-controlled study of chronic myocardial infarction patients receiving intra-myocardial injections of autologous BM-derived mononuclear cells, cell therapy patients had a decrease in summed stress score and increase in left-ventricular (LV) ejection fraction at 3 and 6 months (both statistically significant).^127^ A subsequent study of 87 patients with severe LV dysfunction revealed no statistical differences in LV ejection fraction or size of infarct between placebo and autologous BMNC infusion.^128^ A much smaller study revealed that both autologous BM MNCs and expanded BM MSCs yielded a decrease in myocardial scarring by 3 months, indicating beneficial tissue remodeling.^129^ Similarly, the percutaneous stem cell injection delivery effects on neomyogenesis (POSEIDON) randomized trial comparing allogeneic and autologous MSCs in 30 ischemic cardiomyopathy patients indicated increased functional capacity, quality-of-life and ventricular remodeling as a result of both allogeneic and autologous cell therapy.^64^ Most recently, direct myocardial injection of autologous, expanded BM MSCs resulted in persistent improvements in exercise capacity, Canadian cardiovascular scale (CCS) class score, angina attack frequency and nitroglycerin consumption at 1 year post intervention.^130^

#### Vascular disease

Vascular diseases comprise a wide variety of pathologies, all characterized by some level of blood vessel malfunction or damage. Chronic obstructive pulmonary disease (COPD) is characterized by airway obstruction and loss of functional lung tissue caused by a number of factors including inflammation and deleterious tissue remodeling.^131^ It has been hypothesized that cell therapies could beneficially have an impact on the inflammatory component of COPD and thereby diminish airway constriction and lung tissue loss associated with emphysema.^131^ An early rat model of COPD demonstrated the ability of adipose-derived adult MSCs in combination with a biomaterial to accelerate regeneration of some damaged lung structures.^132^ MSC-conditioned media has been used to reverse cigarette smoke-induced damage in cultured lung fibroblasts; apoptotic death was inhibited, proliferation was increased and extracellular matrix synthesis was restored in response to MSC-conditioned media treatment.^69^ Despite the promising basic science evidence and animal studies, human translation has yet to be optimized. In a 2012 double-blinded study of 62 COPD patients each subject received an infusion of 100 million allogeneic MSCs, resulting in significant reduction of circulating C-reactive protein.^133^ Unfortunately, the treated patients did not experience significant changes in pulmonary function tests or quality-of-life indicators. However, such severely affected patients may require multiple treatments over a longer time frame.

Peripheral artery disease (PAD) is a frequent result of atherosclerosis. Depending upon the size of the compromised blood vessel, it can deteriorate into critical limb ischemia (CLI), leading to pain and eventual loss of tissue/amputation of ischemic limbs.^134^ As the problem is characterized by compromised blood flow, it can be difficult for clinicians to effectively treat PAD or CLI. Even if a graft or cell therapy could be applied to damaged tissue, it would still lack the blood flow necessary to sustain it. Thus, an effective treatment for PAD or CLI must address the issue of ischemia caused by lack of blood flow. It is possible that the trophic effects of MSCs could positively effect the progression of PAD and/or CLI. Multiple studies have already demonstrated the clinical improvements in ankle-brachial index, transcutaneous partial pressure of oxygen, pain and frequency of amputation achieved through administration of BM MSCs to patients with CLI.^135^ A recent study of 13 ‘no-option' CLI patients demonstrated improved blood perfusion in the affected limbs, suggesting cell-induced angiogenesis as a result of intra-arterial BMC infusion.^136^

### MSC in wound care and soft tissue repair

#### Wounds and ulcers

It has been shown that MSCs secrete a wide variety of paracrine factors and are capable of recruiting macrophages. Endothelial lineage cells are recruited by secretion of VEGF, IGF-1, EGF and angiopoietin-1. As each of these factors has an important role in the process, MSCs have been shown to enhance wound healing in general.^34^ Autologous cultured BM MSCs have been applied in a fibrin spray to chronic wounds attributed to skin cancer surgery (*n*=5) or non-healing lower extremity wounds (*n*=8). Cells were applied up to four times. There was a positive correlation between number of cells applied and rate of wound closure (effective ‘dose' of 1–5 million MSC per cm^2^).^137^

#### Burns

Thermal trauma can result in a substantial amount of collateral tissue damage after the initial injury. If this damage progresses, necrotic tissue can provide a perfect incubator for severe bacterial infection.^138^ As such, any intervention capable of speeding the rate of burn wound healing and/or mitigating tissue loss would be clinically advantageous. Because MSCs are capable of such a wide variety of therapeutic actions, they have understandably been investigated for just such a use. A rat model of burn wounds demonstrated a significant decrease in the rate of apoptosis of dermal cells in and around the initial wound when treated with injection of BM MSCs.^139^ In 2005, a patient with 40% skin area I/II-degree burns and 30% area with III-degree burns was treated with allogeneic BM MSCs resulting in mild pain relief and decreased plasmarrhea after 30 min. Formation of necrotic tissue previously observed in the patient was prevented, and skin grafts accepted better than historically similar cases.^140^ In a slightly dissimilar case, a patient with severe radiation burns, for whom standard treatments had failed, received five grafts of autologous, expanded BM MSCs. The patient exhibited a significant decrease in blood C-reactive protein levels for 100 days following MSC therapy. The patient also experienced complete resolution of pain, absence of necrotic tissue and reconstruction of soft tissues of the arm. The authors proposed that anti-inflammatory, trophic and paracrine functions of MSC were responsible for healing the previously chronic burn wound.^141^

### MSC for neural disorders and spinal cord injury

#### Multiple sclerosis and amyotrophic lateral sclerosis

Multiple sclerosis (MS) is an autoimmune neurological disorder characterized by demyelination of axons in the brain and spinal cord, leading to both physical and mental impairment.^142^ Most treatments focus on suppressing the immune system to prevent resultant neurological damage, but immune suppression alone cannot repair existing neurological damage and is untenable for long-term treatment of the disease. Thus, a truly effective treatment for MS requires attenuation of the autoimmune component as well as regeneration of damaged neural components. MSCs administered in a murine model of neural injury have been shown to migrate to the lesion, increase oligodendrocyte lineage cells in the lesion and drive the immune response toward a more beneficial Th1/Th2 balance.^143^ This same response can be recreated by MSC-conditioned medium and has been attributed to the action of HGF secreted by MSCs.^29^ In 2010, a small study demonstrated the safety and potential benefits of MSC therapy for MS in humans; 10 MS patients received autologous, expanded BM MSCs by intrathecal injection. Five out of seven patients showed improvement in Expanded Disability Status Scale at 6 months. Vision and sensitivity tests showed improvement in 5/6 patients at 3 months.^144^ A larger study of 15 MS and 19 amyotrophic lateral sclerosis (ALS) patients receiving one intrathecal injection of autologous, expanded BM MSCs demonstrated safety and initial immunomodulation effects. The administered cells were magnetically labeled, allowing for MRI visualization of their final destination. Imaging revealed the presence of magnetically labeled cells in the meninges, subarachnoid space and spinal cord. Twenty-one out of 34 patients experienced transient fever, possibly due to exposure to residual cell culture or labeling materials. Amyotrophic Lateral Sclerosis Functional Rating Scale score remained stable for 6 months after intervention, and Expanded Disability Status Scale scores improved from 6.7 to 5.9 on average.^145^ Although intrathecal injection is a logical method of administration, intravenous injection of MSCs would decrease the risk associated with cell therapy intervention for MS and/or ALS. Connick *et al.*^146^ demonstrated the feasibility of such a technique. Ten MS patients received intravenous injection of autologous expanded BM MSCs (1.6 million cells per kg). After 10 months, improvement was demonstrated in visual acuity, evoked response latency and increased optic nerve area. No effects were noted on visual field or retinal nerve fiber thickness. As with other cell therapy applications, more studies are necessary to delineate the best mode of administration and cell type, but the current state of cell therapy in MS and ALS warrants enthusiasm and further investigation.

#### Parkinson's disease

Parkinson's disease (PD) is a progressive, degenerative disease caused by loss of dopaminergic cells in the substantia nigra region of the midbrain; it initially manifests in physical impairment and is later accompanied by mental impairment.^147^ Several studies have demonstrated the presence of stem cells in the brain and the ability of some cell types to differentiate into dopaminergic neurons.^148, 149^ Two recent studies have demonstrated the utility of MSC-based therapy in treating PD. In one study, seven patients aged 22–62 years, average disease duration of 14.7 years, received autologous, expanded BM MSC transplants (1 million cells per kg) into the sublateral ventricular zone by stereotaxic surgery. Three patients demonstrated steady improvement in their ‘off' and ‘on' Unified Parkinson's Disease Rating Scale (UPDRS) score. Average improvement ‘off' score was 22.9% from baseline, whereas ‘on' score improved 38% from baseline. Patients also demonstrated improvement in Schwab and England and Hoehn and Yahr scores and symptoms in facial expression/gait/freezing episodes.^150^ In a later study, 12 patients (eight PD and four PD plus system atropy and progressive supranuclear palsy) received transplant of allogeneic BM MSCs (2 million cells per kg). All PD patients showed improvement with average 17.2% improvement of ‘on' score and 31.2% improvement of ‘off' score in UPDRS. Results correlated with progression of the disease before treatment. PD plus patients showed no improvement with treatment.^151^

#### Stroke

Ischemic stroke is caused by mechanisms similar to those of PAD and CLI and thus presents another logical therapeutic target of cell-based therapies. In 2011, 10 patients with acute middle cerebral artery ischemic stroke received 7–10 million autologous BM TNC per kg. Seven out of nine surviving patients achieved a Barthel Index >90. All nine patients shifted down at least a full point on the modified Rankin Scale. Median National Institutes of Health Stroke Scale score went from 13 before treatment to 8 at day 7, and 3 at 6 months.^152^ The results of this initial study combined with those of PAD and CLI studies indicate that cell-based therapies may be effective for treatment of ischemia-related pathologies and should be investigated further.

#### Spinal cord injury

It has been demonstrated that MSCs secrete a variety of factors that influence neurological healing and regeneration, and MSCs may be capable of directly protecting neural cells.^153, 154^ MSCs implanted in a rat spinal cord injury (SCI) model regulated the inflammatory environment of the injury and activated macrophages to change from M1 to M2 type to enhance tissue remodeling and reduce scar tissue in the early stages after injury.^155^ Multiple studies have applied cell therapies to clinical spinal cord injury patients with varying degrees of success. Five human SCI patients were treated with autologous BM mononuclear cell and granulocyte colony-stimulating factor (GCSF), and one patient was treated with GCSF only. Significant motor progress was observed during months 3 through 7 post therapy. Four patients (80%) receiving cells improved from Grade A to Grade C, and one patient improved from Grade A to Grade B. The GCSF-only patient remained at Grade A. The only side effects observed were fever and myalgia associated with the GCSF administration.^156^ A later study applied expanded autologous BM MNCs to 10 Grade A or Grade B SCI patients. Patients received injections of cells at 4 and 8 weeks. Six out of 10 patients improved in motor function scores of the upper extremities, and three of those patients improved in their daily living activities. MRI showed a decrease in cavity size. At the last follow-up (>30 months post intervention), three out of 10 patients showed improvement in motor power and daily activity, as well as significant MRI evidence of beneficial electrophysiological changes.^157^ Non-expanded BM MSCs have been applied at various time points after SCI. ASIA Impairment Scale (AIS) scores increased in 30% of patients receiving cells before 8 weeks post injury. No cysts, infections, hemorrhage or other adverse events were observed.^158^ In a separate study, four patients with cervical SCI (ASIA grade A) were treated with autologous BM concentrate 1 month after injury. After 12 months, two patients progressed to Grade C, one patient progressed to Grade B and one exhibited no progress. None of the patients suffered complications or adverse events.^159^

Thus far, cell therapies as applied to SCI have investigated autologous BM MSCs and the length of time between injury and various cell administration techniques. The missing comparator in this compendium of information is the effect of biomaterial or other enhancements on the success of cell therapy for SCI. Zurita *et al.*^160^ recently demonstrated the ability of PRP gel to enhance neuronal differentiation of MSCs; PRP could also be used to retain undifferentiated MSCs at the site of application and suppress apoptosis of neighboring cells. Only further investigation will determine which, if any, biomaterial, time course of treatment, administration method or cell type/source is most beneficial for SCI treatment; however, the referenced studies alone establish MSC-based cell therapy as a legitimate clinical option for SCI patients.

### MSC for autoimmune disorders

Autoimmune disorders comprise a wide spectrum of maladies whose pathology stems from a fundamental malfunction in which the immune system recognizes self as non-self and erroneously attacks the tissues of the host.^161^ It has previously been established that MSCs can help drive the immune system toward a more favorable TH1/TH2 balance and increase the number of Treg cells.^143^ These effects are favorable in autoimmune diseases, but so too are the anti-inflammatory and protective effects of MSCs. In autoimmune patients, there is a tendency to doubt the efficacy or potency of their MSCs, owing to their ‘malfunctioning' immune system. Though understandable, this doubt is not borne out by experimental investigation, as BM MSCs from autoimmune disease patients are identical to those of healthy patients in their ability to suppress an *in* vitro immune response and proliferate in a CFU-F assay.^162^ Thus, multiple studies have investigated the application of MSCs to autoimmune disorders like rheumatoid arthritis, Crohn's disease and lupus erythematosus. A case series on compassionate-use cell therapy treatments demonstrated the safety and efficacy of autologous, expanded MSCs in multiple autoimmune diseases. One patient with autoimmune inner ear disease and documented severe hearing loss for 3 years recovered normal hearing in one ear and moderate hearing in the other ear at follow-up. Polymyositis, MS, atopic dermatitis, and rheumatoid arthritis patients were also treated with largely successful results.^163^

Human adipose-derived MSCs induced an increase in Treg cells in a murine model of rheumatoid arthritis, demonstrating the feasibility of applying the same principle to human cases of rheumatoid arthritis.^164^

Two separate studies have recently highlighted the applicability of MSC therapies to Crohn's disease. MSCs from refractory Crohn's disease patients and healthy patients performed identically in laboratory experiments, indicating their equivalence. In a nine-patient study with patients receiving two infusion doses of 2 × 10^6^ cells per kg body weight, one-third of patients experienced a clinical improvement of >70 points decrease in Crohn's disease activity index at 6 week follow-up.^165^ Twelve patients with Fistulising Crohn's disease were treated with intrafistular injection of autologous, expanded BM MSC and achieved sustained complete closure of fistula tracks in seven cases and partial closure in three cases. Rectal mucosa healing was observed in all patients, as well as a significant increase in circulating Treg cells.^166^

Several small-scale studies of various types of MSCs were done on systemic lupus erythematosus (SLE), all of which demonstrated some clinical efficacy. Four early-stage SLE patients treated with allogeneic MSCs showed stable disease remission for 12–18 months.^167^ In a later study, two patients with severe SLE received autologous BM MSC; Treg cells increased significantly but did not significantly modify disease state.^168^ The most recent evidence in favor of MSC-based treatment of SLE involves 15 refractory-stage SLE patients who received BM MSCs from blood relatives. Significant improvements were seen in systemic lupus erythematosus disease activity index (SLEDAI) scores, and proteinuria and stabilization of renal function after 12 months.

Graft-versus-host (GVHD) disease is a type of autoimmune disease. It is an extreme complication after BM transplantation in which the grafted immune system attacks the host tissues. If it goes unchecked, GVHD can lead to death. As the immune modulatory effects and safety of MSCs are well documented, they are ideal candidates for a novel and effective GVHD treatment. Mouse models have demonstrated that infusion of MSCs after BM transplantation can dramatically reduce the progression of GVHD. In a compassionate-use study of *ex vivo* cultured adult human MSCs (prochymal) for treatment of grade III and intravenous GVHD, 5/12 patients survived through follow-up at 611 days. Survival expectations for these patients were between 5 and 10% if left to conventional treatment.^169^

## Efficacy, Safety and Regulatory status of MSC

### Autologous versus allogeneic MSC

The majority of *in vivo* studies of MSC therapies utilize allogeneic or syngeneic (genetically similar) donor cells because of the difficulty of extracting cells in a survival surgery in small animals. It has been proposed by the senior author that although all MSCs respond comparably to biochemical stimuli (up- or downregulation of particular proteins), specific response of each of the patients is determined by their genotype.^30, 33^ Considering the complex feedback mechanisms of immunomodulation, minor differences between host and donor cells may have an impact on trophic and anti-inflammatory effects. In addition to potential loss in efficacy, the use of allogeneic cells presents a risk of a host immune response to the donor cells if detected or in reaction to cell culture and preservation reagents in the cell preparation. As described, MSCs are generally considered immune-privileged, but express detectable levels of HLA Class I antigens. Furthermore, if allogeneic MSCs differentiate *in vivo*, their HLA protein expression will be altered and detectable by the host immune system and potentially elicit a host-versus-graft response. In a mouse study comparing immune response with syngeneic versus allogeneic MSCs, allogeneic cells triggered a significant increase in CD8+, natural killer and natural killer T cells compared with animals receiving syngeneic cells.^63^ In the same study, splenocytes isolated from allogeneic MSC recipients demonstrated a significant INF-γ response *in vitro*. Preliminary clinical trials with allogeneic cells have reached similar conclusions. Perin^170^ reported in a dose-escalation study of allogeneic MSCs injected for ischemic and non-ischemic heart failure that 13% of all patients developed donor-specific anti-HLA antibody response after injection, 9% transiently produced anti-HLA antibodies after 1 month and 4% overall (13% of the high or 150 million cell dose group) had persistent anti-HLA antibodies beyond 1 month. The donor-specific anti-HLA antibodies were determined to be against HLA Class I antigens, indicating the host response to the allogeneic cells.

The differences in clinical efficacy between autologous and allogeneic MSCs and the dose effect of either cell type remain poorly understood. In a transendocardial injection study using autologous and allogeneic BM MSCs for patients with ischemic cardiomyopathy, improvements to subjects' Minnesota Living with Hearth Failure Questionnaire scores and six minute walk time were observed only in patients treated with autologous cells.^64^ In a 32-patient clinical trial treating GVHD with a low (2 million MSC per kg) or high (8 million MSC per kg) dose of allogeneic MSCs, there was a 77% effective response to treatment and no differences in efficacy or safety between the doses.^171^ Preclinical large animal studies evaluating ‘mesenchymal precursor cells' (MPCs; Stro-3+ cells isolated from BM) for spinal fusion and treatment of DDD demonstrated decreased efficacy with higher doses of cells based on outcome criteria (fusion score/bone density in fusion study, disc height index/MRI score/histology score in DDD study).^172, 173^ These results foreshadowed the interim results of the same product's Phase II clinical trial for DDD, in which the lower cell dose (6 million MPCs) demonstrated greater efficacy than the higher cell dose (18 million MPCs) and the higher cell dose had a greater incidence of adverse events.^174^ In an ischemic heart failure clinical study, the lowest dose of MPCs (25 million) resulted in the greatest improvement in LV ejection fraction and LV systolic volume, whereas no significant effect was achieved by 75 or 150 million MPC doses at 3, 6 or 12 months.^170^ In comparison, the same authors previously reported that treatment of heart failure with autologous BM mononuclear cells significantly improved Minnesota Living with Hearth Failure Questionnaire and quality-of-life scores in the cell-treatment group versus the control group.^175^ It also was observed in that study that young patients responded more dramatically to autologous cell therapy than older patients. Cumulatively, these findings suggest that if cells are to be treated as a drug, more is not always better and the minimum and maximum effective doses must be determined based on the clinical application. Because of the inherent biological differences in MSC by donor, and where applicable, their manufacturing and delivery processes, defining such doses by indication is a major obstacle for obtaining regulatory clearance of cell products classified as a drug.

### Culture-expanded versus point-of-care autologous MSC

Because of the initial perception of cells as a drug, doses of MSCs in the thousands, millions or billions based on the recipient's body weight were believed to be necessary for a therapeutic effect.^58, 137, 176^ Such doses are not physiologically compatible with point-of-care isolation of MSCs from BM aspirate, and *in vitro* expansion of cells was utilized to generate the required cell numbers in a matter of days or weeks. A detail that is often overlooked in this process is the elimination of other cell types (hematopoietic stem cells, endothelial cells and so on) and the creation of a homogeneous cell population with successive cell culture passages.^98^ The temporal change and convergent cell phenotype may have an impact on the ‘per cell' therapeutic efficacy. In addition, the inclusion of other phenotypes in a heterogeneous population of fresh cells may benefit the modulation and vascularization of the targeted tissue. The trade-offs of greater MSC numbers versus unaltered and heterogeneous cell preparations have not been sufficiently explored in the literature.

### Regulatory oversight of MSC therapies

Most stem cell therapies are regulated by the US Food and Drug Administration (FDA) under 21 Code of Federal Regulation 1271 for human cells, tissues and cellular and tissue-based products (HCT/Ps).^177^ Category I cell products, including whole blood, BM and organ transplants, are not regulated under this statute.^178^ Category II cell and tissue products (‘361 products') that are not dependent on the metabolic activity of living cells are regulated under Section 361 of the Public Health and Safety Act and require the following: minimal manipulation of the cells/tissue, homologous use, not to be combined with a drug or device and are autologous or used in a first- or second-degree blood relative. Category III products (‘351 products'), including those that are dependent on the metabolic activity of living cells are regulated under Section 351 of the Public Health and Safety Act and include cell and tissue products that are cultured or more than minimally manipulated, not intended for homologous use, are combined with a drug or device or are allogeneic. Products containing viable cells from cadaveric BM have been marketed by companies claiming a ‘361 status, and thus have not been cleared by the FDA. Several BM and whole-blood devices that process cells at the point-of-care with not more than minimal manipulation (for example, centrifugation) have been adopted by surgeons to provide therapeutic cell preparations that are compliant with Section 361. Products that require manipulation and/or are allogeneic, including donor cells, embryonic cells and induced pluripotent stem cells, are considered to be ‘351 products and require an investigational new drug exemption by the FDA and a biologics license application on file with the FDA prior to initiating clinical trials.^179^ Strict definitions of ‘minimal manipulation' and ‘homologous use' have not been delineated, but several FDA untitled and warning letters provide examples of products that do not meet those criteria. For example, *in vitro* expansion of autologous BM cells or the isolation of progenitor cells from adipose tissue by *ex vivo* enzymatic or ultrasound treatment was deemed to not meet the requirements for minimal manipulation.^180, 181, 182^ Further guidance by the FDA is required to clear up the ambiguity surrounding point-of-care cell therapies if autologous, minimally manipulated cells from sources outside of blood and BM are to be utilized without an investigational new drug exemption, biologics license application, and clinical trials. Similarly, a designation of nonhomologous use of autologous, minimally manipulated cells processed at the point-of-care for treatment of arthritis, soft tissue wounds and burns, spinal cord injuries, autoimmune diseases and other disorders with few therapeutic options would significantly delay the use of autologous progenitor cell preparations generally accepted to be safe while substantially increasing the cost of therapy.

## Conclusions

Our understanding of what constitutes an MSC, its metabolic activities and therapeutic potential has improved considerably since the initial isolation of colony-forming cells in the 1960s. New insights into the anti-inflammatory and immunomodulatory capacity of MSCs have altered the original dogma of their therapeutic mechanisms and potential *in vivo*. The benefits of heterogeneous cell populations (including hematopoietic stem cells, endothelial progenitor cells, platelets and so on) and limitations of allogeneic MSCs require further preclinical and clinical investigation. On the basis of the preliminary reports of safety and efficacy in several medical specialties, autologous cell therapies, whether they utilize freshly harvested or culture-expanded cells, represent a method to treat conditions that currently are unmet and result in generally poor outcomes or invasive surgery. Further clinical data are necessary, however, to determine the *in vivo* distribution and therapeutic mechanisms of MSCs and to optimize their use as part of a personalized regenerative medicine strategy. This process will require the collaborative efforts of physicians, scientists, industry and regulatory agencies to translate nature's basic regenerative element into the continuum of clinical care.

## Figures and Tables

**Table 1:** MSC and CFU-F concentrations and frequency derived from adult and near-fetal tissues

*Human tissue source*	*Native CFU-F concentration range per ml of fluid/tissue*	*MSC frequency range (CFU-F/10*^*6*^ *nucleated cells)*	*References*
Bone marrow aspirate	109–664^a^	10–83	^91, 183, 184, 185, 186, 187^
Adipose/lipoaspirate	2058–9650	205–51 000	^98, 184, 188, 189, 190^
Dermis	Not reported	74 000–157 000	^89^
Umbilical cord blood	0.06	0–0.02	^184, 185, 191, 192^
Peripheral blood	0	0–2^b^	^185, 192, 193^
Synovial fluid	4–14	2–250	^92, 194^
Amniotic fluid	3	9.2	^195^

a Based on average of 8 × 10^6^ nucleated cells per ml bone marrow aspirate.^183^

b Occurance of CFU-F in peripheral blood requires systemic treatment with GCSF.

**Table 2:** Anti-inflammatory mechanisms of MSCs

*Target cell*	*Mechanism*	*Primary effect*	*Secondary effect*
Dendritic cells	PGE2/direct contact	↓ TNF-α, IL-12, differentiation and activation	Impairs effect on resting NK cells ↓ T-cell proliferation ↓ INF-γ by T_H_1 cells^a^
	PGE2, IL-6, IL-8 and SDF-1	↑ IL-10	↑ IL-4 by T_H_2 cells^a^
Immature Dendritic cells	PGE2	↑ IL-10	↑ Treg production, ↑ IL-10 by Treg cells
T cells (CD4+, helper T cells)	PGE2, IDO, HGF, TGF-β1 and NO	↓ CD4+ T-cell proliferation by S-phase entry block and Go/G1 phase arrest Inhibits T-cell functions	↓ B-cell proliferation ↓ Ig antibody production by B cells
	IL-10	Inactivate T_H_1 cells	
T cells (CD8+, cytotoxic T cells)	sHLA-G5	↓ cytotoxicity	
Treg cells	IL-10	↑ Treg production ↑ IL-10 by Treg cells	
	sHLA-G5	↓ Treg differentiation	
B cells	PGE2, HGF, TGF-β1, IDO, NO and PD-L1	↓ B-cell proliferation by Go/G1 phase arrest ↓ Ig antibody production by B cells, ↓ B-cell chemotaxis	
NK cells	PGE2, IDO, sHLA-G5, HGF, TGF-β1	↓ INF-γ ↓ NK cell proliferation ↓ cytotoxicity	
Monocytes	PGE2	↓ Monocyte proliferation by Go/G1 phase arrest ↓ Monocyte difference to DC	
Macrophages	IL-6	↓ TNF-α	
	TSG-6	↓ NF-kB	↓ TNF-α and IL-1 ↓ MMP synthesis
	PGE2	Converts M1 (pro-inflammatory) type to M2 (anti-inflammatory) type macrophages	↓ IL-10 ↓ IL-12 ↓ TNF-α
Neutrophils	IL-6	↓ respiratory burst ↓ apoptosis	
No specific target	VEGF	Pro-angiogenic	Increased nutrient, O_2_ and waste transport
	IL-1Ra	Antagonizes IL-1	
	sTNF-R	Inhibits TNF-α production	↓ T-cell proliferation, ↓ INF-γ by T_H_1 cells^a^

Abbreviations: HGF, hepatocyte growth factor; HLA, human leukocyte antigen; IDO, indoleamine 2,3-dioxygenase; IL-1Ra, IL-1 receptor antagonist; INF, interferon; MMP, matrix metalloproteinase; NF-κB, nuclear factor kappa-light-chain-enhancer of activated B cell; NK, natural killer; NO, nitrous oxide; PD-L1, programmed cell death ligand-1; PGE2, prostaglandin 2; SDF-1, stromal cell-derived factor-1; sTNF-R, soluble TNF-α receptor; TGF, transforming growth factor; TNF, tumor necrosis factor; TSG, tumor necrosis alpha-stimulating gene; VEGF, vascular endothelial growth factor.

a Promotes T_H_1→T_H_2 T-cell transition.
